# Ginkgo leaf extract and dipyridamole injection as adjuvant treatment for acute cerebral infarction: Protocol for systemic review and meta-analysis of randomized controlled trials

**DOI:** 10.1097/MD.0000000000014643

**Published:** 2019-02-22

**Authors:** Yongyong Liu, Xuqi Wu, Zhenwei Yu

**Affiliations:** a Kaihua Hospital of Traditional Chinese Medicine, Qu Zhou; b Huzhou Third Municipal Hospital, Huzhou; c Sir Run Run Shaw Hospital, College of Medicine, Zhejiang University, Hangzhou, Zhejiang, China.

**Keywords:** alternative medicine, ginkgo biloba, ischemic stroke, traditional chinses medicine

## Abstract

**Background:**

Acute cerebral infarction (ACI) is one of the most commonly seen cerebral vascular disease and the current therapy options are not satisfied. Ginkgo leaf extract and dipyridamole injection (GDI) is widely used as adjuvant therapy for ACI. However, there is no systemic review and meta-analysis published regarding the efficacy and safety of GDI. Herein, we describe the protocol of a proposed study aims to systemically evaluate the efficacy and safety of GDI in ACI patients.

**Methods:**

Five electronic databases (Medline, EMBase, Cochrane database, China National Knowledge Infrastructure, and Wanfang database) will be searched up to February 28, 2018. Randomized controlled trials (RCTs) meet the eligibility criteria will be identified and included. Data synthesis will be run using RevMan software after the data extraction and risk of bias assessment of included studies. The primary outcomes of this study are effective rate and adverse event rate.

**Results:**

This study will provide a high-quality synthesis of RCTs on the efficacy and safety of GDI as an adjuvant therapy in the treatment of ACI.

**Conclusion:**

This systemic review and meta-analysis will provide high quality evidence to evaluate GDI as adjuvant therapy in patients with ACI.

**Registration:** PEROSPERO CRD42018107112

## 1 Introduction

Acute cerebral infarction (ACI), also referred as ischemic stroke, is one of the most commonly seen cerebral vascular disease and poses a considerable threat to millions of people in China.^[1,2]^ ACI is always resulted from cerebral blood circulation disorder, hypoxia and ischemia, and can lead to neurological impairment, even death.^[3]^ Acute treatment of ACI aims to restore the brain tissue perfusion, improve microcirculation, protect cerebral cells, control cerebral edema and so on.^[4]^ Conventional western medicine (WM) therapeutics includes recombinant tissue-type plasminogen activator, anticoagulants, fibrinolytic agents, antiplatelet agents. However, the drug therapies may carry more than minimal risk and the current therapy options are not satisfied.^[5]^

Many scholars hold the opinion that adjuvant therapy, such as traditional Chinese medicine, will provide benefit for patients with ACI.^[6]^ Ginkgo leaf extract and dipyridamole injection (GDI) is a compound preparation, which consists of ginkgo flavone glycosides, terpene lactones and dipyridamole. Ginkgo biloba is a traditional Chinese herb and widely used in brain disorder treatment from the ancient time.^[7]^ Ginkgo extract can protect the neurons against reactive oxygen species, calcium overload, and improves damaged neural energy metabolism.^[8–10]^ Ginkgo extract can also reduce infarction size by improving neurological function.^[11]^ As the clinical evidence accumulates, GDI is widely used for ischemic disorders in China.^[12]^

In the hierarchy of evidence-based medicine, systemic reviews of high quality randomized controlled trials are considered as the best evidence regarding specific healthcare interventions. However, there is no systemic review and meta-analysis published regarding the efficacy and safety of GDI as an adjuvant therapy in the treatment of ACI. Here we have written this protocol and planned to carry out a systemic review and meta-analysis to assess the clinical effect and safety of GDI in the ACI treatment.

## 2 Methods

This protocol follows the Preferred Reporting Items for Systemic Review and Meta-Analysis Protocol (PRISMA-P) statement.^[13]^ And this work was registered in PROSPERO (CRD42018107112). Ethical approval is not needed because this study only involves previous studies.

### 2.1 Eligibility criteria

#### 2.1.1 Type of study

Only randomized controlled trials can be included. Observation studies, case reports and case series are excluded.

#### 2.1.2 Participants

Patients diagnosed with ACI. There is no restriction regarding diagnose standard used in the original study.

#### 2.1.3 Intervention

ACI patients treated with conventional WM regimen and GDI as adjuvant treatment. There is no restriction regarding conventional WM treatment regimen.

#### 2.1.4 Comparison

ACI patient treated with the same conventional WM regimen as intervention group in the same original study.

#### 2.1.5 Language

Studies published in English and Chinese will be included. Studies published in other languages will also be included if the reviewers obtain required information by contacting the original author or getting help form translators.

### 2.2 Information source

The following databases will be searched electronically: Medline, EMBase, Cochrane database, China National Knowledge Infrastructure (CNKI), and Wanfang database. The last search day will be February 28, 2019.

### 2.3 Search strategy

The electronic search will be run using a combination of following items: Ginkgo, Ginkgo biloba, Ginkgo Leaf, Ginkgo biloba Leaf, Extract, Dipyridamole, Cerebral infarction, Cerebral ischemia, Cerebral thrombosis, Brain infarction, Brain ischemia, Ischemic stroke, and Ischemia cerebral stroke. Manual search will be done if relevant literature are found in the included studies.

The literature search will be done by 2 reviewers (YYL and XQW) independently and cross checked. Any inconsistency will be solved by a third reviewer (ZWY).

### 2.4 Study selection and data extraction

The searched studies will be managed using a reference manage software, NoteExpress (V3.2.0.7222, AEGEAN technology, Beijing). Two reviewers (YYL and XQW) will review and select studies according to eligibility criteria independently, and then crosscheck. Any inconsistency will be solved by a third reviewer (ZWY).

The following items of included studies will be extracted by 2 reviewers (YYL and XQW) independently: first author, published year, study duration and religion, number and age of participants, severity of illness, intervention of experimental group and control group, outcomes and adverse events. If required data is not published in the included literature, the reviewer will contact the corresponding author of original study to request additional information by E-mail. Any inconsistency will also be solved by a third reviewer (ZWY).

### 2.5 Outcomes

#### 2.5.1 Primary outcome

Effective rate. Clinical cure and clinical improvement defined by the original author of included studies are regarded as effective in this review.

Adverse event rate. Adverse events and reactions caused by GDI during the study follow-up.

#### 2.5.2 Secondary outcomes

Score of neurological deficits measured by National Institute of Health Stroke Scale and Nerve Deficiency Scale. Quality of life measured by Barthel Index.

### 2.6 Risk of bias in included studies

The Cochrane risk of bias assessment tool will be used to evaluate the risk of bias in included studies.^[14]^ There are 7 items included: random sequence generation, allocation concealment, blinding of participants and researchers, incomplete outcome data, selective reporting bias, and other bias. Each item will be categorized into “Low Risk”, “Unclear” or “High Risk”. The judgment will be given by 2 reviewers (YYL and XQW) independently and any inconsistency will also be solved by a third reviewer (ZWY).

### 2.7 Data synthesis

Data will be synthesized using Review Manager 5.3 software (Copenhagen: The Nordic Cochrane Centre, The Cochrane Collaboration, 2014). The odds ratio and 95% confidential interval will be calculated for primary outcomes. The mean difference and 95% confidential interval will be calculated for continuous outcomes. Heterogeneity among studies will be investigated using the I^2^ test before data synthesis (I^2^ > 50% is defined to indicate significant heterogeneity). The Mantel-Haenszel fixed effect model will be used when no significant heterogeneity exists among studies; otherwise, a random model will be used. Subgroup analysis will be carried out base on following items:

1. kind of WM combination used,
2. measurement stages during the treatment course.

Sensitivity analysis will be run using a leave-one-out method. Publication bias will be evaluated by funnel plots if the number of studies included in the analysis is more than 10.

### 2.8 Summary

The results of the main outcomes will be summarized using the grading of recommendations assessment, development, and evaluation approach.^[15]^

## 3 Discussion

This protocol presents the methodology of a systemic review and meta-analysis evaluating the clinical efficacy and safety of GDI as an adjuvant therapy in the treatment of ACI. To the best of our knowledge, this would be the first study analyzing the data from RCTs regarding this topic. We will run the systemic review according to protocol and reported it according to PRIMSA guidelines. And we believe that the result of this review will be helpful for the treatment of ACI. The limitation is that the credibility of this review will be affected by the quality of included studies.

## Author contributions

YYL and ZWY had the original idea for a meta-analysis. YYL and XQW designed the protocol. YYL and ZWY reviewed the search strategy. YYL and XQW drafted the protocol. ZWY registered in PROSPERO. YYL is the guarantor of the protocol. All authors read and approved the final version.

**Conceptualization:** Yongyong Liu, Zhenwei Yu.

**Funding acquisition:** Zhenwei Yu.

**Methodology:** Yongyong Liu, Xuqi Wu, Zhenwei Yu.

**Supervision:** Yongyong Liu.

**Writing – original draft:** Yongyong Liu, Xuqi Wu.

**Writing – review & editing:** Yongyong Liu, Zhenwei Yu.
